# Editorial for the Special Issue “Advances in Microbial and Plant Biotechnology”

**DOI:** 10.3390/microorganisms14092005

**Published:** 2026-09-10

**Authors:** Svetlana Veselova, Igor Maksimov

**Affiliations:** Institute of Biochemistry and Genetics, Ufa Federal Research Centre, Russian Academy of Sciences, Prospekt Oktyabrya, 71, 450054 Ufa, Russia; maksimov@ufaras.ru

**Introduction**

In the modern scientific literature, plants and their microbiomes are considered holobionts—unified evolutionary and functional systems comprising the host and its symbiotic microorganisms [1,2,3]. A beneficial microbiome transforms inaccessible mineral compounds into bioavailable forms, fixes atmospheric nitrogen, participates in protection against abiotic and biotic stresses, and produces phytohormones regulating plant morphogenesis [2,3]. This holobiont-based perspective opens up new possibilities for microbial biotechnology—a dynamically developing field that harnesses the metabolic and genetic potential of microorganisms (bacteria, fungi, yeasts). The key products of these technologies in agriculture are biofertilizers (microbial inoculants enhancing nutrient bioavailability and nitrogen fixation) and biopesticides (antagonistic microorganisms and their metabolites) [4,5,6]. Promising *Bacillus* sp., *Pseudomonas* sp., and *Streptomyces* sp. strains synthesize various metabolites (siderophores, lipopeptides, antibiotics), effectively suppressing phytopathogenic fungi [7,8], while the combination of bacteria with elicitors and biostimulants (non-nutrient substances or microorganisms with the ability to promote plant growth and health) enhances both protective and growth-promoting effects [9,10]. This paves the way for the development of next-generation biocontrol agents with triple action: pathogen protection, growth stimulation, and enhanced resistance to abiotic stresses. According to recent reviews, microbial biotechnologies are regarded as a cornerstone for achieving sustainable agroecosystem development [11,12].

To highlight recent advances and progress in the field of microbial and plant biotechnology, the Special Issue “Advances in Microbial and Plant Biotechnology” was established. This Special Issue brings together the latest innovations in research on plant–microbe interactions and identifies new perspectives and directions in this actively developing and highly relevant field of modern biotechnology.

This Special Issue comprises twenty original research articles contributed by research groups from East and South Asia, Europe, South America, and North Africa, demonstrating the global impact of this research area. All studies are dedicated to the role of microorganisms in plant life—ranging from pathogenesis to symbiosis and defense—and are united by a common goal: the search for environmentally safe and effective solutions for plant protection and yield enhancement. All articles employ cutting-edge technologies, including sequencing, real-time PCR, CRISPR/Cas9, gene expression analysis, metabolomics (high-performance liquid chromatography–mass spectrometry (HPLC-MS)), bioinformatics, and machine learning, ensuring high reliability and robustness of the obtained data. The contributions to this Special Issue cover various directions of microbial biotechnology, including the study of microbial communities and pathogenic microorganisms, the design of biopesticides and biofertilizers, as well as genetic engineering of microorganisms and applied biotechnology. For improved readability and conceptual clarity, all articles have been grouped according to their thematic focus.

**Biotechnology of Microbiome Management: From Diagnostics to Therapy of Agroecosystems**

The study of plant-associated microbial communities is one of the most rapidly advancing fields in contemporary microbiology and biotechnology, as evidenced by five contributions to this Special Issue [5,13,14,15,16]. The application of sequencing-based approaches—targeting 16S rRNA genes and the internal transcribed spacer (ITS) region of ribosomal DNA—enables a transition from descriptive taxonomy to functional analysis of microbial communities as an integral component of plant holobionts. These technologies not only facilitate comprehensive taxonomic profiling of bacterial and fungal communities across diverse agroecosystems [5,13] but also allow tracking of community dynamics in response to various stressors, including nitrogen fertilization [14], antibiotic exposure [15], and siderophore application [16].

Gharsallah et al. [13] performed the first large-scale molecular identification of the olive grove microbiome in Tunisia, thereby addressing a critical gap in knowledge regarding the structure and spatiotemporal dynamics of microbial communities in Mediterranean olive agroecosystems. The taxonomic composition—dominated by *Bacillus*, *Penicillium*, *Aspergillus*, and *Cladosporium*—was shown to depend on geographic location, substrate type, and sampling year. Importantly, the presence of well-characterized biocontrol agents and plant growth-promoting microorganisms (PGPM) (*Bacillus*, *Pseudomonas*, and *Trichoderma*) among the identified genera offers a promising basis for future functional studies and the development of biopreparations for olive disease and pest management.

Nitrogen fertilizers are fundamental to modern agricultural intensification; however, the accumulation of their derivatives—ammonia, nitrous oxide, and nitrates—in the environment is recognized as one of the foremost global ecological dangers. Two articles in this Special Issue, by Huang et al. [14] and Safronova et al. [5], underline the critical importance of maintaining nitrogen balance in agroecosystems for sustainable crop production. The former addresses the negative impact of excess nitrogen on the soil microbiome, while the latter focuses on symbiotic nitrogen fixation. Huang et al. [14] demonstrate that even short-term application of nitrogen fertilizer leads to nitrate accumulation in the surface soil layer and the disruption of the soil microbial community. Safronova et al. [5] provide the first evidence that co-inoculation of commercial rhizobial strains with strains from relict legumes—harboring unique genes—significantly enhances the nitrogen-fixing efficiency of symbiosis in common vetch (*Vicia sativa*) and red clover (*Trifolium pratense*), opening up new prospects for the development of microbial consortia that reduce dependence on nitrogen fertilizers. Collectively, Safronova et al. [5] and Huang et al. [14] offer a systems-level perspective on the role of nitrogen in agroecosystems: biological nitrogen fixation and controlled fertilizer application represent two complementary pillars of sustainable agriculture.

The functionality of microbial communities in the rhizosphere and endosphere of plants is determined by intermicrobial interactions mediated by secondary metabolites, including siderophores and antibiotics, which govern the structure, composition, and resistance of microbial associations. Two articles in this Special Issue—by Shen et al. [16] and Danilova et al. [15]—address the impact of microbial metabolites on microbial community structure and function. Employing a metagenomic approach (16S rRNA and ITS sequencing), Shen et al. [16] demonstrated for the first time that siderophores produced by *Trichoderma* and *Beauveria* fungi do not merely suppress the pathogen *Ralstonia solanacearum*, but systematically restructure the rhizosphere microbial community of diseased plants, stimulating soil enzymatic activity, which opens up a novel strategy for restoring the healthy ecological balance of the ecosystem. In a complementary study, Danilova et al. [15] demonstrated the vertical migration of antibiotic resistance genes (*tet*(A) and *tet*(X)) from the substrate into roots and subsequently into leaves. This finding underscores the potential risks of antibiotic resistance dissemination through the food chain and highlights the urgent need to prevent its entry into the food supply.

All five studies [5,13,14,15,16] address a central question: what defines a healthy agroecosystem, and what factors disrupt it? Together, they demonstrate the necessity of shifting the management paradigm from combating individual pathogens to managing the microbiome as an integrated whole.

**Evolutionary Biotechnology of Pathogens: Genetic Diversity, Adaptation, and Molecular Diagnostics**

The study of pathogenic microorganisms, their genetic diversity, and evolutionary plasticity is critically important for the development of effective plant protection strategies under conditions of agricultural intensification and climate change. Two articles in this Special Issue—by Wu et al. [17] and Akosah et al. [18]—provide fundamental insights into the evolutionary dynamics of pathogenic microorganisms and establish a scientific foundation for transitioning from reactive plant protection measures (response after problem emergence) to systemic molecular monitoring and predictive management. Wu et al. [17] present the first evidence of the high genetic diversity of *Calonectria pseudoreteaudii*, the causal agent of Eucalyptus leaf blight, and underscore that effective breeding for resistant varieties is unreachable without a comprehensive understanding of pathogen population structure.

Fungicide resistance in phytopathogenic fungi poses a serious threat to agriculture. *Fusarium oxysporum* is a major pathogen involved in potato diseases, and phenylpyrroles and azoles are commonly employed for its management; however, data on their efficacy against this pathogen remain limited. Akosah et al. [18] elucidate, for the first time, the molecular mechanisms underlying *F. oxysporum*’s resistance to two major fungicide classes: resistance to penconazole (azoles) is mediated by overexpression of the sterol-14α-demethylase gene (*CYP51a*), while resistance to fludioxonil (phenylpyrroles) is associated with overexpression of the histidine kinase gene (*HK1*). Notably, the observation of pathogen growth stimulation at low fludioxonil concentrations—a hormetic effect—raises serious concerns regarding the continued use of this compound. This study fills a critical gap in understanding the evolutionary plasticity of *F. oxysporum* and provides a foundation for transitioning from empirical fungicide application to molecularly informed monitoring and the development of alternative control strategies, including biocontrol.

The research by Wu et al. [17] and Akosah et al. [18] changes the paradigm of plant protection by demonstrating that effective management of fungal pathogens necessitates the integration of population-genetic monitoring and molecular markers of resistance.

**Biotechnology of Multifunctional Microbial-Based Biopreparations**

The development of multifunctional biopreparations for the protection of plants against pathogens, pests, and abiotic stresses constitutes a strategic direction in contemporary microbial biotechnology. Bacteria belonging to the genera *Pseudomonas*, *Streptomyces*, and *Bacillus* exhibit a unique combination of traits—production of antimicrobial compounds, induction of systemic resistance, promotion of plant growth, and reinforcement of physical barriers (biofilm formation and lignification)—which collectively enable the design of biopreparations with complex action. Eight articles in this Special Issue are dedicated to exploring these aspects [6,7,8,9,10,19,20,21].

Two studies by Sidorova et al. [7] and Boykova et al. [8] were dedicated to the identification and characterization of active metabolites produced by bacteria of the genera *Pseudomonas* and *Streptomyces*, employing the highly sensitive HPLC-MS methodology. Sidorova et al. [7] found that the antagonistic activity of novel *Pseudomonas* sp. strains against *Fusarium graminearum*, dependent on the metabolomic profile of phenazines and lipopeptides, and growth promotion do not necessarily co-occur within a single strain, thus providing a rational basis for the development of synthetic microbial consortia. Boykova et al. [8] conducted a comprehensive characterization of *Streptomyces* sp. strain P-56, demonstrating for the first time its ability to produce the macrotetrolide antibiotic nonactin, which exhibits a remarkably broad spectrum of biological activities, and to combine these biocontrol properties with multiple plant growth-promoting traits, making it a highly promising candidate for the development of next-generation multifunctional biopreparations.

Next, two articles by Yarullina et al. [9] and Javed et al. [19] focused on the activation of plant defense responses through combination bacterial strains and elicitors—compounds produced by microorganisms that trigger a cascade of defense responses in the host. Javed et al. [19] demonstrated that the protein elicitor secreted by the entomopathogenic fungus *Verticillium lecanii* 2, PeVL1, activates systemic resistance in rice against the rice leaf roller *Marasmia ruralis* through the induction of hormonal signaling pathways. Furthermore, the PeVL1 elicitor modified the physical properties of the leaf surface, creating unfavorable conditions for pest colonization and reducing the fecundity of the pest. Yarullina et al. [9] demonstrated that the combined application of *Bacillus subtilis* (strains 26D and 11VM) and conjugates of chitin with hydroxycinnamates confers synergistic control of *Phytophthora infestans* on potato. This work demonstrates the priming–trigger mechanism of a combined biopreparations (bacteria prime the plant’s defense system, while chitosan acts as a trigger to activate it), expanding the conventional concept of a “biopesticide”—now redefined as a tool that “educates” plants to defend themselves, thereby ensuring more durable and sustainable disease protection.

The studies by Sharipova et al. [20] and Maslennikova et al. [21] focus on the physicochemical mechanisms through which beneficial microorganisms enhance plant stress tolerance—encompassing both structural modification of plant cell walls and the formation of protective biofilms in the rhizosphere. Sharipova et al. [20] address a critical gap in understanding biofilm formation regulatory mechanisms in plant-associated *Bacillus subtilis*, involving a regulatory network of extracellular proteases and external factors including temperature, pH, and metal ions. Maslennikova et al. [21] provide the first evidence that the endophytic strain *B. subtilis* 10-4 accelerates lignification of cell walls of wheat roots under cadmium stress, thereby creating a physical barrier against heavy metal ions and limiting their translocation to aboveground tissues. Collectively, the studies by Sharipova et al. [20] and Maslennikova et al. [21] demonstrate that the protective effect of PGPM involves both biochemical regulation and structural modulation—a dual functionality that is critically important for the development of biopreparations targeting multi-component stress in agroecosystems. This fundamentally expands our understanding of PGPM modes of action: they not only “treat” the plant but also “fortify” its structural integrity.

Bacteria that combine metabolic, hormonal, and stress-protective mechanisms of growth promotion represent a particularly promising strategy for enhancing crop productivity under the challenges of climate change. The studies by Suleimanova et al. [6] and Feoktistova et al. [10] investigated the potential of developing multifunctional biofertilizers capable of promoting plant growth through the simultaneous improvement of mineral nutrition and protection against environmental stresses, thereby contributing to enhanced agroecosystem resilience. Suleimanova et al. [6] demonstrated that *Pantoea brenneri* strains possess a unique capacity for phosphate solubilization from diverse recalcitrant organic and inorganic sources, achieving efficiencies of up to 87% through the production of extracellular enzymes or by releasing organic acids, which was confirmed by the presence of the corresponding genes in the genome of the bacteria. Genes responsible for phosphate binding, import, and transport were also identified. These properties stimulate the growth of wheat and potato. Feoktistova et al. [10] reported a synergistic effect of combined *Pseudomonas plecoglossicida* 2,4-D and humic substance application on root growth and nitrogen nutrition in wheat under water deficit conditions by modulating the plant hormonal balance. The key stress-protective mechanisms of this combination were a reduction in abscisic acid (ABA) levels and an elevation of the cytokinin content. Collectively, the studies by Suleimanova et al. [6] and Feoktistova et al. [10] demonstrate that bacteria can enhance plant growth through metabolic and hormonal mechanisms, thereby enabling the more efficient utilization of available resources and enhanced adaptation to stressful environments.

Taken together, eight of the research articles presented in this Special Issue [6,7,8,9,10,19,20,21] establish a solid foundation for the development of next-generation biopreparations. These biopreparations are designed not merely to eliminate pathogens or pests, but rather to holistically promote plant health—stimulating growth, reinforcing immunity, improving mineral nutrition, and enhancing stress tolerance. The principal breakthrough lies in the demonstration that it is fundamentally feasible to integrate the functions of fertilizer, pesticide, and stress protectant within a single product. This capability fundamentally transforms the plant protection paradigm, shifting the conceptual framework from “combating enemies” to “caring for plant health.”

**Plant and Microbial Engineering Through Epigenetics and CRISPR**

The implementation of precise genome editing tools and the deepening understanding of molecular regulatory mechanisms mark a transformative shift in microbial biotechnology—moving from empirical screening and random mutagenesis toward the rational design of strains with tailored properties, thereby opening new frontiers for agribiotechnology. Two studies by Danilova et al. [22] and Rumyantsev et al. [23] were dedicated to developing tools for the precise improvement of bacterial strain characteristics and elucidating their mechanisms of action at the genomic level. Danilova et al. [22] employed CRISPR/Cas9 to inactivate antimicrobial peptide genes in *B. pumilus* 3-19, demonstrating that targeted genome editing can enhance the properties of biocontrol agents or, alternatively, identify key effector molecules for the development of synthetic formulations. In another study, Rumyantsev et al. [23] provided the first evidence of the regulation of RNA interference components (encompassing Argonaute (*AGO*) and Dicer-like (*DCL*) genes) and microRNAs expression in wheat plants in response to treatment with the endophytic strain *B. subtilis* 26D during aphid (*Rhopalosiphum padi*) infestation. The authors proposed a model in which microbial signals, transduced through hormonal cascades, induce genome reprogramming at the level of small RNAs, in turn orchestrating the synthesis of defense proteins, secondary metabolites, and modulate cellular redox status, representing a fundamentally new dimension in our understanding of immune “priming.” Collectively, these studies establish a robust platform for the development of “intelligent” biopreparations capable of exerting precise, predictable, and targeted effects on host plants.

**Applied Biotechnology: From Laboratory to Field**

A central challenge in contemporary biotechnology is bridging the gap between fundamental research and its practical implementation in agricultural production. Three articles in this Special Issue—by Topilina et al. [24], Balseca et al. [25], and Ullah et al. [4]—are distinguished by their strong applied orientation, proposing economically accessible agrotechnologies. Each study incorporates optimization—of cultivation protocols [24], medium composition [25], or agronomic practices [4]—and validates the efficacy of the proposed solutions under realistic conditions, including vegetation trials, industrial cultivation, or field experiments. Topilina et al. [24] demonstrated that inoculation of highbush blueberry microclones with the novel mycorrhizal fungus *Leptodophora* sp. BR2-1 significantly improves plant adaptation to non-sterile conditions by stimulating root and shoot development. Balseca et al. [25] employed response surface methodology (RSM) to optimize the culture medium for the cyanobacterium *Arthrospira platensis* (spirulina), replacing expensive commercial components with low-cost urea. Ullah et al. [4] proposed an agronomically sound strategy to reduce the chemical burden on rice agroecosystems—partial substitution of urea with biochar. This approach not only improves soil physicochemical properties (pH, organic carbon content) but also stimulates ammonia-oxidizing bacteria (AOB), thereby enhancing nitrogen use efficiency and rice yield.

The developed technologies facilitate the replacement of costly imported components with locally available alternatives [25], the utilization of waste or by-products (biochar, grain substrate) to enhance soil fertility [4,24], and the resolution of specific production challenges—from microclone adaptation to the production of high-value metabolites. Collectively, these studies extend beyond fundamental knowledge generation to offer practical instruments for transitioning toward ecologically sustainable and economically efficient agricultural systems.

**Future Perspectives**

A detailed analysis of the articles in this Special Issue reveals several key directions that will define the future trajectory of microbial biotechnology in agriculture. The future lies not in the development of individual biopreparations, but in the creation of intelligent agroecosystems managed through the microbiome. The next-generation strategy envisions a transition to ecologically sustainable and resource-efficient systems, with the following core components: rapid diagnostic profiling of the soil microbiome to detect pathogens and beneficial microorganisms; selection of tailored “microbial cocktails” optimized for specific crops, cultivars, soil types, and prevailing weather conditions; integration of host genetic resistance with the application of synthetic microbial consortia and their bioactive metabolites; and rigorous surveillance of antibiotic resistance dissemination and environmental contamination.

**Conclusions**

Collectively, the studies presented in this Special Issue reflect a global trend in contemporary plant and microbial science: a fundamental shift from chemical-intensive to biologically based models of agroecosystem management. The authors investigate complex networks of interactions among pathogens, beneficial microorganisms, and plants at both molecular and ecological scales, employing cutting-edge approaches including genomics, transcriptomics, metabolomics, and genetic engineering. The next-generation biopreparations developed—whether live microbial strains or their purified metabolites—not only suppress diseases but also promote plant health and restructure microbiome composition, contributing to more sustainable and environmentally compatible agricultural systems. The significance of these articles lies in their translation of fundamental research into actionable strategies for addressing pressing global challenges—food security, environmental pollution, and resource depletion.

## Data Availability

No new data were created or analyzed in this study. Data sharing is not applicable to this article.
